# Multicystic Dysplastic Kidney Disease: An In-Utero Diagnosis

**DOI:** 10.7759/cureus.37786

**Published:** 2023-04-18

**Authors:** Rajas Chaubal, Sindhu Chandra Pokhriyal, Amol Deshmukh, Uma Gupta, Nitin Chaubal

**Affiliations:** 1 Obstetrics and Gynaecology, Jaslok Hospital & Research Centre, Mumbai, IND; 2 Internal Medicine, One Brooklyn Health, New York, USA; 3 Obstetrics and Gynaecology, Thane Ultrasound Centre, Mumbai, IND; 4 Internal Medicine, Interfaith Medical Center, New York, USA

**Keywords:** disease, mcdk, ultrasound, kidney, cystic

## Abstract

Multicystic dysplastic kidney (MCDK) is a congenital cystic kidney disease that can be incidentally seen during the antenatal ultrasound. The condition is most commonly asymptomatic. The clinical presentation is usually characterized by multiple small cysts or a single dominating cyst in the fetal kidney depending on the type of MCDK. Most cases undergo spontaneous involution, and complications like hypertension, infection, and malignancy are rare. We present the case of a young Primigravida who was diagnosed to have a fetus with unilateral MCDK in the second trimester and further followed up later in pregnancy as well as four months postnatally. The pregnancy was unremarkable, but for the diagnosis of MCDK in the second trimester; the infant was doing well at the four-month follow-up. Pre-natal ultrasound and MRI are able to diagnose MCDK reliably. Conservative management and follow-up is currently the most common protocol used to manage MCDK.

## Introduction

Multicystic dysplastic kidney (MCDK) is a common pediatric renal condition that is characterized by multiple non-communicating cysts in the kidney with little or no functioning renal parenchyma [1,2]. It is the commonest cause of renal cystic disease in neonates [2]. MCDK is a developmental disorder that occurs when the urinary tract is obstructed during embryogenesis [3,4]. The obstruction leads to an abnormal metanephric-mesenchymal differentiation, which causes MCDK [4,5]. These non-functional cysts most often undergo complete agenesis and are therefore commonly asymptomatic [2]. However, they can also be associated with complications or malformations such as vesicoureteric reflux (VUR), cryptorchidism, and other genital abnormalities [2,3].

MCDK can be unilateral or bilateral. Unilateral MCDK is more common than bilateral MCDK and is more commonly seen in male fetuses [2]. The incidence of unilateral MCDK is roughly 1:4000, with a predisposition for the left kidney [1]. Bilateral MCDK is more likely to be linked to non-renal problems and is incompatible with life [1,2].

MCDK can be sporadic, non-familial, or rarely associated with a genetic syndrome [5,6]. MCDK can, in some cases, have a genetic etiology and has also been found to be associated with syndromes such as Meckel-Gruber syndrome, Joubert syndrome, and Zellweger syndrome [6].

Whenever it is symptomatic, MCDK presents with symptoms like flank pain, recurrent UTI (secondary to VUR), hypertension, and/or a palpable mass [2]. It may present as an incidental finding in the early years of life when infants undergo ultrasounds for other indications. With the rise in the number of comprehensive prenatal anatomy ultrasounds performed around 20 weeks of pregnancy, diagnosis is frequently also made in the fetus while it is still in utero [4,7].

Complete spontaneous resolution of MCDK can be seen in as many as 60% of cases [6]. Infection, hypertension, and malignancy of MCDK are some of the less frequent complications of MCDK [8]. It can be associated with contralateral renal abnormalities like VUR, pelviureteric junction obstruction, vesicoureteric junction obstruction, renal agenesis, and hypoplasia [3]. Here, we present the case of unilateral MCDK that was diagnosed in the second trimester and followed up till four months postpartum.

## Case presentation

**Figure 1:**
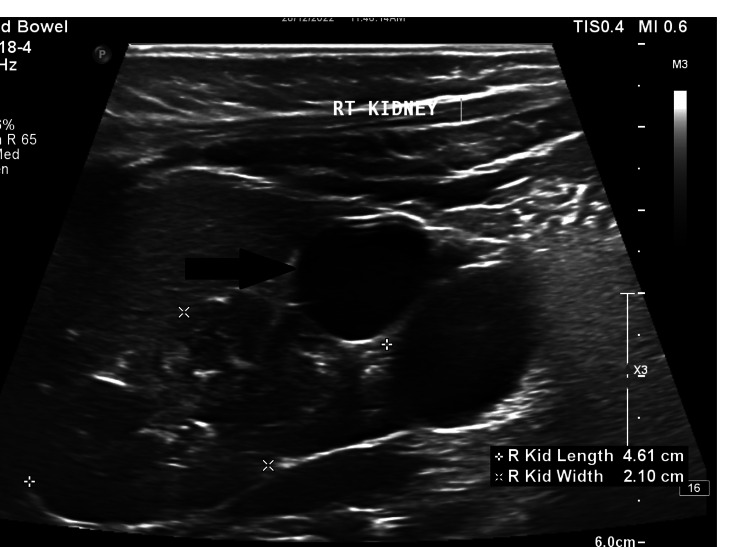
Enlarged right fetal kidney measuring 4.6 cm x 2.1 cm

**Figure 2:**
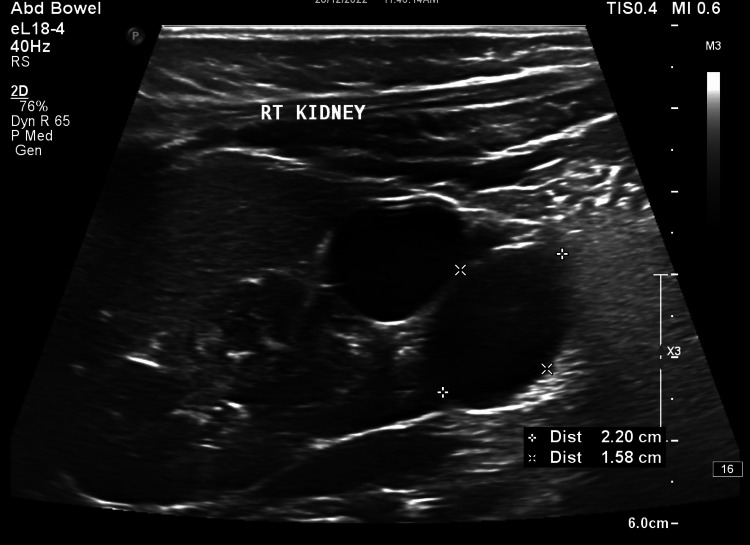
Enlarged right fetal kidney with non-communicating cysts in the lower pole of the kidney

**Figure 3:**
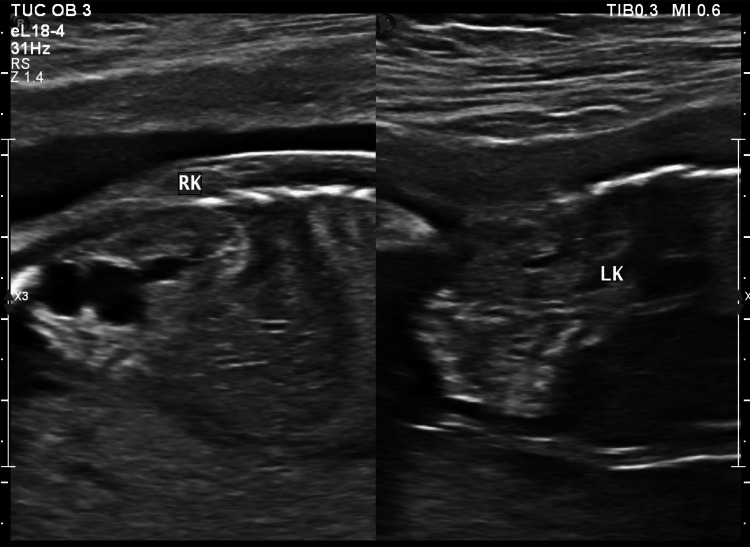
Sagittal section of the fetal abdomen showing the enlarged right fetal kidney and the normal-sized contralateral (left) kidney

A 20-year-old primigravida with a history of spontaneous conception and a normal nuchal translucency scan at 13 weeks was referred to our hospital at 19 weeks gestation for a comprehensive anatomy scan. Pregnancy had been unremarkable so far with no complaints. A fetal ultrasound study revealed a bulky right fetal kidney, 4.6 cm x 2.1 cm in size, with calyceal dilatation in the lower pole of the kidney (Figure 1). Findings were suspicious for the possibility of a duplex moiety due to incomplete fusion of the upper and lower poles of the collecting system. The amniotic fluid was noted to be normal. Due to limited resources, a fetal MRI could not be done, and the shared decision of a follow-up scan at 24-25 weeks was made. The repeat ultrasound at 24 weeks showed non-communicating cystic areas in the lower pole of the right kidney and a provisional diagnosis of MCDK was made (Figure 2). The contralateral kidney was evaluated and appeared normal (Figure 3). The patient went on to have an uneventful antenatal course and a spontaneous normal vaginal delivery of a male neonate at 39 weeks gestation. The infant was followed up in the combined fetal medicine and pediatric urology clinic four months after birth. He was noted to be asymptomatic and a follow-up ultrasound showed multiple cysts in the lower pole of the right kidney suggestive of focal multicystic dysplastic changes with a normal contralateral kidney. Blood pressure (BP) was normal and no associated genitourinary abnormalities were present. The prognosis was explained to the parents and a follow-up appointment with a pediatric urologist was arranged.

## Discussion

MCDK is the second most common cause of abdominal mass in neonates [1]. While unilateral MCDK has a far better prognosis, is typically asymptomatic, and involutes over time, bilateral MCDK is mostly incompatible with life [9]. Various factors have been proposed as the causative factors of MCDK. These include genetic factors, intrauterine infections, and teratogens [10]. MCDK can occasionally be linked to nonrenal problems, the most frequent of which are genital, cardiac, and musculoskeletal issues. Additionally, MCDK has been linked to genetic disorders such as Waardenburg syndrome, Turner syndrome, and Trisomy 21 [6]. Many cases of MCDK are diagnosed before birth, sometimes as early as the fifteenth week of pregnancy when many tiny cysts begin to appear [7]. In our case, the unilateral MCDK was first diagnosed in-utero, on an ultrasound done at 19 weeks. Clinical presentation varies from large dominant cystic masses to multiple cystic masses occupying a localized part of the kidney [3,6]. The non-functional renal parenchyma is classically fibrous with a lobulated appearance that consists of numerous internal cysts of various sizes and shapes [6,7]. Two main types of MCDK have been described. These include the pelvic-infundibular variant, which was also seen in our case, is more common, and is associated with multiple small clusters of non-communicating cysts with atresia of the renal pelvis and ureter. The second type is the hydronephrotic-obstructive type, which is seen as a dominant cyst in the renal pelvis on scans [6].

In order to rule out any connections with the ureter and among themselves, real-time imaging is very helpful [11]. Various MRI sequences, including steady-state free precession (SSFP), T2-weighted imaging, and diffusion-weighted imaging, are used to illustrate MCDK and the affiliated abnormalities clearly. A mercaptoacetyltriglycine (MAG3) or diethylenetriamine pentaacetate (DTPA) scan is also particularly helpful to check for concomitant obstructive uropathy in the hydronephrotic variant of MCDK [6,11]. Up to 60% of cases can undergo complete spontaneous involution and although resolution is most common in the first three years of life, this may take up to 10 years to happen [6,11].

Conservative management with follow-up is therefore the approach followed by most clinicians [7,9]. Serial ultrasonography is used to follow up on the patients until spontaneous involution occurs [7,9]. In our patient, the infant was followed postnatally, and as he continued to be asymptomatic, the plan is to do a close follow-up with serial ultrasounds. MCDK can be associated with various genitourinary anomalies such as VUR, pelviureteric stenosis and obstruction, ureterocele, and cryptorchidism [3,8]. Of these, VUR is by far the most common complication, occurring in between 16% and 21% of the contralateral kidneys of affected patients. Patients with a urinary tract infection or ultrasonographic signs of VUR, like urinary tract dilatation, may benefit from further workup using a voiding cystourethrography (VCUG) [3,11].

The risk of hypertension in MCDK is less than in the average population [12]. The rate of malignant transformation of MCDK is minimal [10]. This low risk of malignant transformation of an MCDK also raises questions about the usefulness of preventative surgical removal [7]. Recurrent UTI, sudden or progressive increase in the size of the kidney, and worsening or persistent flank pain are some of the indications of surgery [1].

Prenatal sonography can thus reliably diagnose MCDK, and the prognosis is affected by the presence of extrarenal and contralateral renal abnormalities [10].

## Conclusions

At present, MCDK is most often diagnosed antenatally. Ultrasound findings of a cluster of non-communicating cysts in the kidney and/or a dominant cyst in the renal pelvis should first trigger the evaluation of the contralateral fetal kidney. Unilateral MCDK with a normal contralateral kidney carries an excellent prognosis. The next step should be to rule out other congenital genitourinary anomalies like VUR or cryptorchidism. Given the low risk of hypertension and malignancy, the current approach involves follow-up with serial ultrasounds and blood pressure monitoring. Surgery is only considered in limited clinical situations such as recurrent UTI, abdominal pain, and a sudden or progressive increase in kidney size.
